# Dataset of polycyclic aromatic hydrocarbon recoveries from a selection of sorbent tubes for thermal desorption-gas chromatography/mass spectrometry analysis

**DOI:** 10.1016/j.dib.2020.105252

**Published:** 2020-02-08

**Authors:** M. Ariel Geer Wallace, Joachim D. Pleil, Donald A. Whitaker, Karen D. Oliver

**Affiliations:** a Center for Environmental Measurement and Modeling, Office of Research and Development, U.S. Environmental Protection Agency, 109 T.W. Alexander Drive, Research Triangle Park, NC, 27711, USA; b Center for Computational Toxicology and Exposure, Office of Research and Development, U.S. Environmental Protection Agency, 109 T.W. Alexander Drive, Research Triangle Park, NC, 27711, USA

**Keywords:** Polycyclic aromatic hydrocarbon (PAH), Thermal desorption, Gas chromatography-mass spectrometry (GC/MS), Sorbent tubes, Carbograph, Tenax, XRO-440

## Abstract

This dataset contains raw area counts and percent recoveries of polycyclic aromatic hydrocarbon (PAH) standards desorbed from selected sorbent tubes and analyzed using thermal desorption-gas chromatography/mass spectrometry (TD-GC/MS). The results of this study were published in the article “Recovery and reactivity of polycyclic aromatic hydrocarbons collected on selected sorbent tubes and analyzed by thermal desorption-gas chromatography/mass spectrometry” in Journal of Chromatography A [1]. The sorbent tubes studied include stainless steel Carbograph 2TD/1TD, glass quartz wool-Carbograph 2TD, inert-coated stainless steel Carbograph 2TD, glass and stainless steel Tenax TA, PAH (chemical weapons), and glass and stainless steel XRO-440 sorbent tubes. Tables listing the experimental conditions, TD methods, and types of sorbent tubes are included in the manuscript. Data for experiments, including the investigation of incomplete desorption of PAHs from Carbograph 2TD/1TD and XRO-440 sorbent tubes, the comparison of PAH recoveries from three different TD methods, the analysis of PAH breakthrough from sorbent tubes, the investigation of the effect of heat on PAH percent recovery from sorbent tubes, and the formation of reaction products during PAH loading and desorption are included in Appendix A. These data can be used to guide sorbent tube selection for PAH analyses in future studies.

**Table:** Specifications Table

Subject area	*Chemistry*
More specific subject area	*Analytical chemistry, environmental science*
Type of data	*Tables and spreadsheets*
How data was acquired	*A PerkinElmer 650 TurboMatrix ATD system and an Agilent* 6890N GC *coupled to an Agilent 5975 inert XL MS (ATD-GC/MS) instrument*
Data format	*Raw and analyzed data*
Experimental factors	*Polycyclic aromatic hydrocarbon standards were diluted in methanol and loaded onto selected sorbent tubes with different packing materials and under varied conditions, including loading time and temperature.*
Experimental features	*Sorbent tubes loaded with polycyclic aromatic hydrocarbon standards were desorbed using the ATD-GC/MS instrument under varied experimental conditions. The percent recoveries of eight priority polycyclic aromatic hydrocarbons were determined and compared with those obtained using other experimental conditions.*
Data source location	*Research Triangle Park, NC, USA*
Data accessibility	*The data are available within this article and in the attached*Appendix A.
Related research article	*Wallace, M.A.G., Pleil, J.D., Whitaker, D.A., and Oliver, K.D. Recovery and reactivity of polycyclic aromatic hydrocarbons collected on selected sorbent tubes and analyzed by thermal desorption-gas chromatography/mass spectrometry. Journal of Chromatography A****2019****, 1602: 19–29.*

**Table:** 

**Value of the Data** • Researchers can use these data as a guide for sorbent tube selection for PAH analyses. • Researchers can compare the percent recoveries of PAHs desorbed from Carbograph 2TD/1TD, Tenax, XRO-440, and PAH (chemical weapons) to PAH recoveries from additional sorbent tubes in future analyses. • Researchers can use these data for TD-GC/MS method optimization for PAH desorption and analysis.

## 1 Data

The presented experimental data are shown in a supplementary Microsoft Excel file, Appendix A. This Excel file was created using Microsoft Office 365 ProPlus version 1808. These data were used to support the results and conclusions presented in Geer Wallace et al. 2019 [1]. Appendix A contains a cover page with the title, author lists, and author affiliations. A data description tab is included to explain the layout of the spreadsheet. Experimental data are included in nine tabs: Incomplete Desorption, Carbograph Method 1, Carbograph Method 2, Carbograph Method 3, Method Optimization, Breakthrough, Heat, Additional Sorbent Tubes, and Reaction Products. These tab names correspond to the names of the experiments that were performed.

The tab for “Incomplete Desorption” in Appendix A includes the raw area counts and percent recoveries of PAHs desorbed from stainless steel Carbograph 2TD/1TD and glass XRO-440 sorbent tubes. The date the sample was acquired, data file name, sample name, and miscellaneous information (including the concentration of PAHs spiked, the loading date, loading time, and whether or not heat was used during loading) are included in this tab. The tab also includes the raw area counts and percent recoveries of the PAHs after the sorbent tubes were desorbed a second time without tube conditioning. The calculated fractions of PAHs remaining on the sorbent tubes after the second desorption are shown.

The “Carbograph Method 1”, “Carbograph Method 2”, and “Carbograph Method 3” tabs in Appendix A include the raw area counts, average area counts (with standard deviation and coefficient of variation), percent recovery, and average percent recovery (with standard deviation and coefficient of variation) of PAHs desorbed from stainless steel Carbograph 2TD/1TD sorbent tubes using three different desorption methods. The data file names, PAH concentrations, heat temperature, loading time, and sample descriptions are provided. The summarized percent recovery data for acenaphthylene, acenaphthene, fluorene, phenanthrene, anthracene, fluoranthene, and pyrene from these three experiments are located in the “Method Optimization” tab. This tab includes the Carbograph thermal desorption method number (1, 2, or 3) and the desorption flow rate of each method (20, 40, or 60 mL/min).

The “Breakthrough” tab in Appendix A contains the raw area counts and percent recoveries for PAHs desorbed from stainless steel Carbograph 2TD/1TD and glass XRO-440 sorbent tubes after 30 s vs. 4 min loading times without heat. The average percent recoveries, standard deviations, and coefficients of variation (CVs) for each PAH from this experiment are also included. Metadata, including the date the sample was acquired, the data file name, data file path, sample name, and miscellaneous information (concentration of PAHs loaded, loading time, and loading date) are also provided.

The “Heat” tab in Appendix A contains the raw area counts and PAH recoveries for heated sample loading vs. room temperature sample loading for stainless steel Carbograph 2TD/1TD and glass XRO-440 sorbent tubes. Data for the following conditions are included: 2.0 ng PAHs loaded for 30 s without heat, 2.0 ng PAHs loaded for 30 s with heat, 2.0 ng PAHs loaded for 4 min without heat, and 2.0 ng PAHs loaded for 4 min with heat. Metadata, including the date the sample was acquired, the data file name, data file path, sample name, and miscellaneous information (concentration of PAHs loaded, loading time, whether or not heat was used, and loading date) are also provided. The temperature of heated loading was approximately 127 °C.

The “Additional Sorbent Tubes” tab in Appendix A includes the sorbent tube name, manufacturer, and catalog number for the nine sorbent tubes that were compared: stainless steel Carbograph 2TD/1TD, glass quartz wool-Carbograph 2TD, inert-coated stainless steel Carbograph 2TD, quartz wool manually added to stainless steel Carbograph 2TD/1TD, glass XRO-440, stainless steel XRO-440, glass Tenax TA, stainless steel Tenax TA, and inert-coated stainless steel PAH (chemical weapons) sorbent tubes. The data file number, sample description, tube number, raw area counts, average area counts (with standard deviation and CV), percent recovery, and average percent recovery (with standard deviation and CV) are listed for all replicate injections of PAHs on each sorbent tube. In all experiments, 2.0 ng of the PAH standard was loaded for 30 s with heat except for the quartz wool manually added to stainless steel Carbograph 2TD/1TD, which was loaded for 4 min.

The “Reaction Products” tab in Appendix A includes the sorbent tube name, manufacturer, and catalog number for the nine sorbent tubes that were compared: stainless steel Carbograph 2TD/1TD, glass quartz wool-Carbograph 2TD, inert-coated stainless steel Carbograph 2TD, quartz wool manually added to stainless steel Carbograph 2TD/1TD, glass XRO-440, stainless steel XRO-440, glass Tenax TA, stainless steel Tenax TA, and inert-coated stainless steel PAH (chemical weapons) sorbent tubes. The data file number, sample description, tube number, and raw area counts, percent recovery, and average percent recovery (with standard deviation and CV) are listed for 9-fluorenone and 9,10-anthracenedione. For all experiments, 2.0 ng of the PAH standard was loaded for 30 s with heat except for the quartz wool manually added to stainless steel Carbograph 2TD/1TD, which was loaded for 4 min.

## 2 Experimental design, materials, and methods

The eight types of sorbent tubes selected for PAH analysis and their conditioning temperatures, conditioning times, and catalog numbers are listed in Table 1 of “Recovery and reactivity of polycyclic aromatic hydrocarbons collected on selected sorbent tubes and analyzed by thermal desorption-gas chromatography/mass spectrometry” [1]. These sorbent tubes were supplied by either Markes International (Gold River, CA, USA) or PerkinElmer Life & Analytical Sciences (Shelton, CT, USA). Sorbent tubes were conditioned using a TC-20 tube conditioner (Markes International) except for XRO-440 sorbent tubes, which were conditioned using a desorption cycle on the PerkinElmer automated thermal desorber (ATD).

Standards were prepared from a 2000 ng/μL Restek Corporation (Bellefonte, PA, USA) PAH standard mix with 16 compounds (catalog no. 31011, lot no. A099403). PAHs were diluted in HPLC grade methanol (Fisher Scientific, Hampton, NH, USA) to prepare 1.0 and 2.0 ng/μL concentrations. Standards were loaded onto sorbent tubes in 1 or 2 μL volumes by spiking the PAHs directly onto the quartz wool (for glass tubes) or by injecting the liquid into the tube using a loading port (for stainless steel and inert-coated tubes) in a steady stream of helium at a flow rate of 50 mL/min for either 30 s or 4 min. Sorbent tubes were loaded at either room temperature or when the loading port was heated to approximately 127 °C. Additional details regarding PAH standard loading procedures can be found in Geer Wallace et al. [2].

A PerkinElmer (PE) 650 TurboMatrix ATD system and an Agilent 6890N GC coupled to an Agilent 5975 inert XL MS instrument were used for sample analyses. All TD methods employed a purge time of 5 min, a column flow rate of 2 mL/min, an outlet split of 6 mL/min. The valve and transfer line temperatures were set at 270 and 290 °C, respectively, and the trap temperature was set from 10 to 385 °C with a 10 min hold. A Rxi-5Sil MS capillary GC column with a 5 m Integra Guard column, 0.25 mm ID, 30 m length, and 0.25 μm film thickness (part no. 13623-124) was purchased from Restek Corporation. Research grade helium gas (99.9999%) and ultra zero air were obtained from Airgas (Morrisville, NC, USA). The GC oven temperature was held at 35 °C for 2 min and increased to 190 °C at 6 °C/min, then increased to 310 °C at 28 °C/min and held for 8 min. The quadrupole, ion source, and transfer line temperatures were 176, 290, and 290 °C, respectively. Scan spectra were collected at a rate of 2ˆ2 and the SIM/scan method option was used. Ions were monitored from 35 to 300 *m/z*. Naphthalene, acenaphthylene, acenaphthene, fluorene, phenanthrene, anthracene, fluoranthene, and pyrene were targeted in the SIM method.

Chromatographic peaks were integrated using ChemStation software version D.02.00. Percent recoveries were calculated using Equation (1) with the assumption that naphthalene was fully recovered in each experiment.

(1) Percent Recovery = (area counts of PAH/area counts of naphthalene) × 100

In the analysis of incomplete desorption, the percentage of PAHs remaining on the sorbent tubes after the first desorption cycle was determined using Equation (2).

(2) % PAHs = (PAH area in second desorption/area of PAHs spiked) × 100
